# Anti-Aging Potentials of Methylene Blue for Human Skin Longevity

**DOI:** 10.1038/s41598-017-02419-3

**Published:** 2017-05-30

**Authors:** Zheng-Mei Xiong, Mike O’Donovan, Linlin Sun, Ji Young Choi, Margaret Ren, Kan Cao

**Affiliations:** Department of Cell Biology and Molecular Genetics, University of Maryland, College Park, MD 20742 USA

## Abstract

Oxidative stress is the major cause of skin aging that includes wrinkles, pigmentation, and weakened wound healing ability. Application of antioxidants in skin care is well accepted as an effective approach to delay the skin aging process. Methylene blue (MB), a traditional mitochondrial-targeting antioxidant, showed a potent ROS scavenging efficacy in cultured human skin fibroblasts derived from healthy donors and from patients with progeria, a genetic premature aging disease. In comparison with other widely used general and mitochondrial-targeting antioxidants, we found that MB was more effective in stimulating skin fibroblast proliferation and delaying cellular senescence. The skin irritation test, performed on an *in vitro* reconstructed 3D human skin model, indicated that MB was safe for long-term use, and did not cause irritation even at high concentrations. Application of MB to this 3D skin model further demonstrated that MB improved skin viability, promoted wound healing and increased skin hydration and dermis thickness. Gene expression analysis showed that MB treatment altered the expression of a subset of extracellular matrix proteins in the skin, including upregulation of elastin and collagen 2A1, two essential components for healthy skin. Altogether, our study suggests that MB has a great potential for skin care.

## Introduction

Skin is the largest and the most visible organ of the human body. Aged skin is biologically characterized by the flattening of the dermal-epidermal junction and a general atrophy of the extracellular matrix (ECM) with disorganized and reduced collagen and elastin^1, 2^. There are two different types of skin aging, intrinsic and extrinsic, caused by physiological and environmental factors, respectively^1–3^. The intrinsic skin aging reflects the naturally occurring changes in the skin as we age, and is clinically manifested as fine wrinkles on the dry skin. Extrinsic skin aging is an accelerated form due to exposure of the skin to sunlight and/or air pollution and is phenotypically demonstrated as dry, rough, pigmented and abraded skin especially in the face and hands. Although they present with different clinical features, both types of skin aging are due in part to the oxidative damage caused by free radicals.

By balancing free radical production and antioxidant neutralization, cells normally keep reactive oxygen species (ROS) at low levels^4^. As we age, a combination of the accumulation of ROS and the reduced ROS scavenging capacity leads to increased oxidative stress that results in the damages of macromolecules in organs. When the skin is routinely exposed to stressful factors from the environment, such as UV radiation, smoke, and pollutants, an elevated number of free radicals will be produced which accelerate skin aging^2^. The over-abundance of ROS decreases collagen synthesis and increases collagen breakdown by stimulating matrix metalloproteinase (MMP) expression, eventually causing the alterations of the dermal matrix^5, 6^. Based on this ROS theory, an effective approach to delay skin aging is to externally supply antioxidants through skincare products to either suppress the production or neutralize the excess free radicals^6^.

Methylene blue (MB), a century-old drug first synthesized in 1876, has been used in clinical medicine for treatment of diverse aliments, e.g. methemoglobinemia, malaria, vasoplegia, septic shock, cancer chemotherapy, and Alzheimer’s disease^7–9^. MB is a diaminophenothiazine with a low redox potential of 11 mV. This property allows for efficient cycling between the oxidized form MB and the reduced form MBH2, which facilitates electron transport in the mitochondria and reduces mitochondrial superoxide production. It also induces the expression of mitochondrial complexes II & IV^9, 10^. Additionally, MB is highly permeable in biological membranes because of its solubility in both water and organic solvent, which permits it to freely enter the intracellular compartments like mitochondria, lysosomes and the nucleus^10–12^.

MB has recently drawn attention not only for its neuroprotective effects on treating Alzheimer’s disease^7, 9^ but also for its anti-aging properties^10, 13, 14^. Previous studies have shown that upon the treatment with MB, normal fibroblasts have displayed increased cellular lifespan, improved cell proliferation and reduced expression of p16, a biomarker of physiological aging^9, 10^. In addition, MB extended the life span of female mice by 6% when included in food^14^. Recently we have shown that MB at nanomolar concentration rescued abnormal nuclear and mitochondrial phenotypes, stimulated cell proliferation and delayed senescence in skin fibroblasts from patients with Hutchinson-Gilford progeria syndrome (HGPS, progeria), a rare genetic disorder of accelerated aging^13^.

Based on these observations, we speculate that MB may effectively protect skin from oxidative stress and delay skin aging. To test this idea, here, we investigated the anti-aging effects of MB in human skin using 2D primary dermal fibroblasts and reconstructed 3D human skin models.

## Results

### MB is a more potent ROS scavenger than NAC, MitoQ, and mTEM

To evaluate the effectiveness of MB as an antioxidant, we first compared the effects of MB treatment with the effects of three other popular ROS scavengers, specifically, N-Acetyl-L-cysteine (NAC), MitoQ, and MitoTEMPO (mTEM). (Supplemental Tables 1 and 2). NAC is a widely used, general ROS scavenger that acts as a precursor of glutathione synthesis and stimulates certain enzymes involved in glutathione regeneration^15^. MitoQ is a modified coenzyme Q10 with a selective accumulation in mitochondria^16^. mTEM is a mitochondrial-targeting superoxide dismutase mimetic that possesses superoxide and alkyl radical scavenging properties^17, 18^. In order to evaluate the effects of each antioxidant, primary skin fibroblasts from a middle-aged normal individual and an HGPS patient were treated for 4 weeks. Mitochondrial ROS (indicated by MitoSOX), the main resource of the total cellular ROS, was then measured through FACS analysis. HGPS cells were used as an accelerated model for normal aging since they share many features in common with physiological aging^19^ (Supplemental Tables 1 and 2).

**Figure 1:**
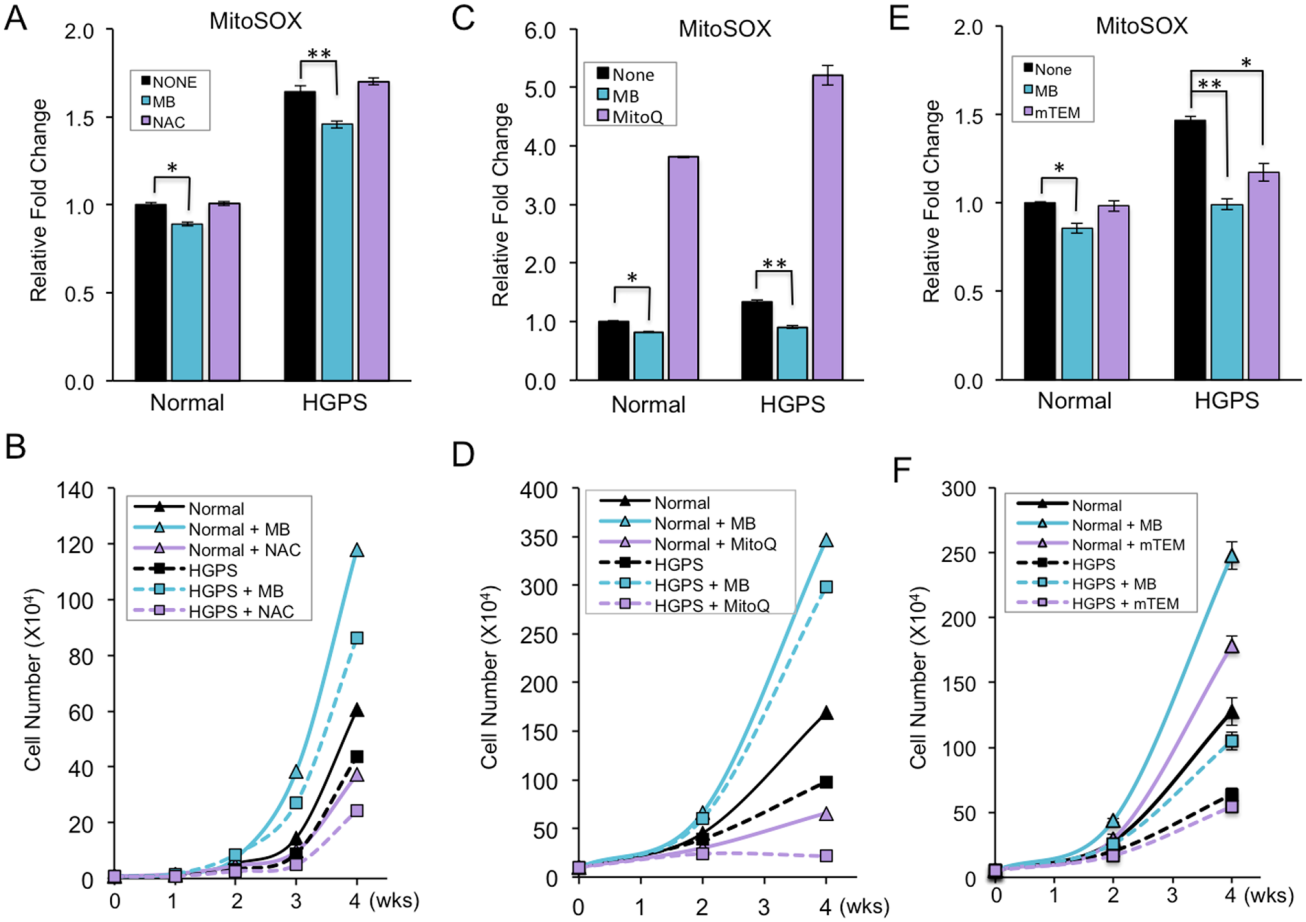
MB is a more potent ROS scavenger than NAC, MitoQ and mTEM. (**A**) Comparison of mitochondrial specific superoxide (MitoSOX) levels in normal and HGPS fibroblasts treated with vehicle, 100 nM MB or 100 μM NAC for four weeks. (**B**) Growth curves of normal and HGPS fibroblasts during the four-week treatment with vehicle, 100 nM MB or 100 μM NAC. (**C**) Comparison of MitoSox levels in normal and HGPS fibroblasts treated with vehicle, 100 nM MB or 100 nM MitoQ for four weeks. (**D**) Growth curves of normal and HGPS fibroblasts during the four-week treatment with vehicle, 100 nM MB or 100 nM MitoQ. (**E**) Comparison of MitoSox levels in normal and HGPS fibroblasts treated with vehicle, 100 nM MB or 100 nM mTEM for four weeks. (**F**) Growth curves of normal and HGPS fibroblasts during the four-week treatment with vehicle, 100 nM MB or 100 nM mTEM. (*p < 0.05, **p < 0.01).

NAC was supplemented in the culture medium at a concentration of 100 μM according to a previous publication^20^. In contrast to the anti-aging effects of MB, long-term treatment with NAC did not reduce mitochondrial ROS level and appeared to delay cell proliferation in both normal and HGPS cells (Fig. 1A and B). To our surprise, treatment with MitoQ at 100 nM, as suggested by previous studies^16, 21^, did not reduce but drastically increased mitochondrial ROS level (Fig. 1C). Additionally, MitoQ treatment did not promote but inhibited cell proliferation in both normal and HGPS cells (Fig. 1D). Treatment of mTEM at 100 nM showed moderate ROS scavenging effects on HGPS cells (Fig. 1E). It also moderately promoted normal cell proliferation but failed to stimulate HGPS cells (Fig. 1F). Amongst all four tested anti-oxidants, MB was the most effective in reducing mitochondrial ROS and promoting skin cell proliferation (Fig. 1A–F, Supplemental Table 2).

### MB reduces aging signs in old skin cells

**Figure 2:**
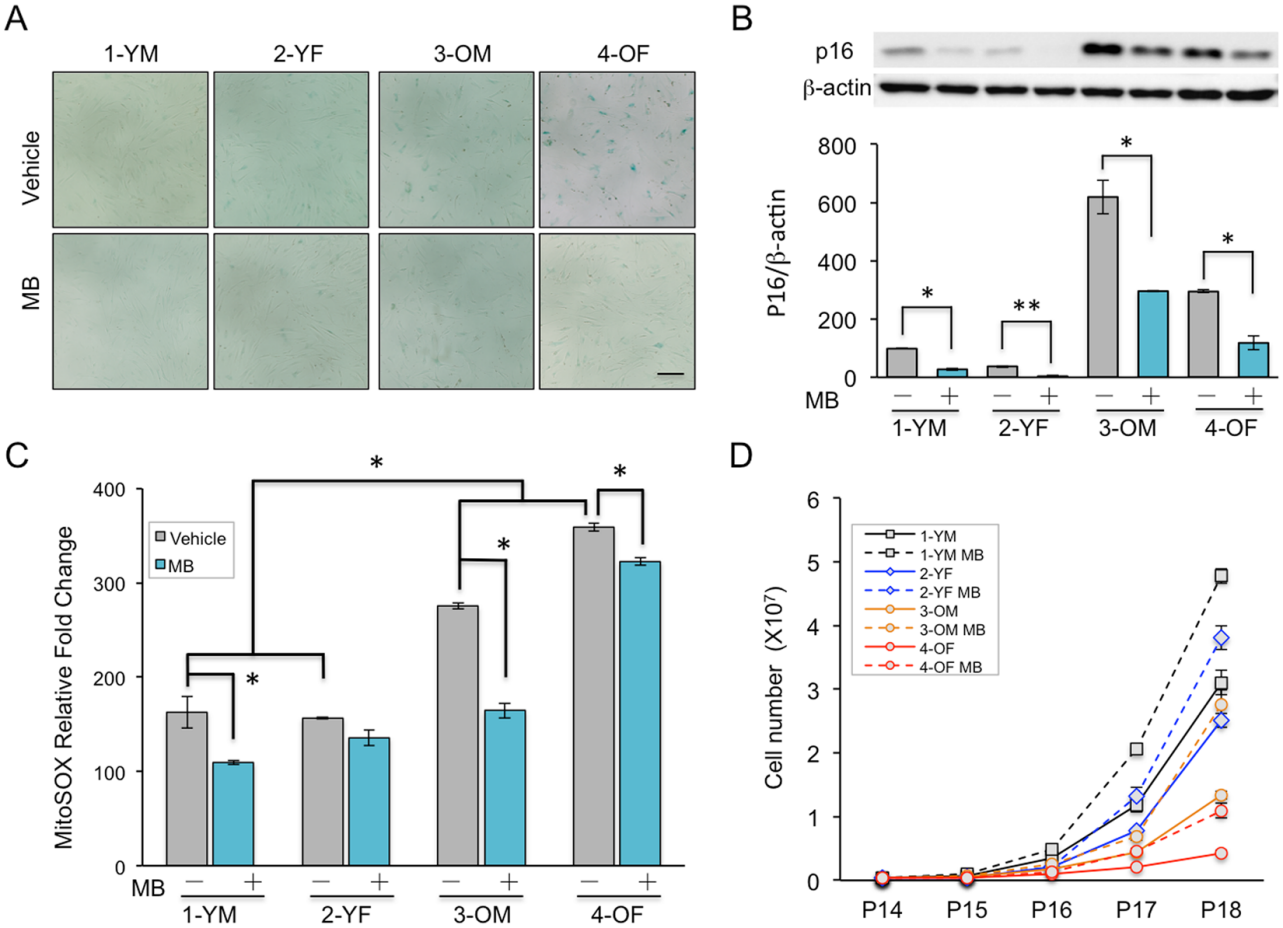
MB reduces aging signs in old skin cells. (**A**) The images of senescence-associated β-galactosidase (SA-β-gal) staining in two young (1-YM & 2-YF) and two old (3-OM & 4-OF) human skin fibroblast lines that were treated with vehicle or 100 nM MB for four weeks. Scale bar = 200 μm. (**B**) Western blotting analysis with an anti-p16 antibody in the two young (1-YM & 2-YF) and two old (3-OM & 4-OF) cells after four weeks of treatment with vehicle or MB at 100 nM. (**C**) Relative fold change of mitochondrial superoxide (MitoSOX) levels in the two young and two old fibroblasts after four weeks of treatment with vehicle or MB 100 nM. (**D**) Growth curves for vehicle- (solid lines) or MB- (dashed lines) treated young and old fibroblasts during the four-week treatment of each drug. (*p < 0.05, **p < 0.01).

Next, we asked whether MB treatment could delay or reverse aging phenotypes from skin cells derived from old individuals. Two old dermal fibroblast lines from individuals over 80 years old (3-OM & 4-OF) and two young skin fibroblast lines from individuals below 30 years old (1-YM & 2-YF) were selected for MB treatment (Supplemental Table 1). The old fibroblasts, particularly the 4-OF cells, showed severe senescence phenotypes. At the molecular level, 3-OM and 4-OF lines demonstrated increased SA-β-gal signals and p16 expression, two widely used senescence biomarkers, in comparison to those in the young cells (1-YM & 2-YF) (Fig. 2A and B). FACS analysis revealed much higher levels of mitochondrial ROS in 3-OM and 4-OF than control cells 1-YM and 2-YF (Fig. 2C). Furthermore, the old cells proliferated much more slowly than the young cells (Fig. 2D, solid lines). Particularly, the 4-OF cells stopped growing towards the end of the experiment at passage 18 (Fig. 2D).

After growing cells in culture medium supplementing 100 nM MB for four weeks, it was evident that the aging-related phenotypes were significantly reduced in the old cell lines 3-OM and 4-OF. MB treatment effectively reduced SA-β-gal signals and decreased the expression of p16 in 3-OM and 4-OF cells (Fig. 2A and B). In addition, MB treatment decreased the elevated MitoSOX in old cell lines, especially in the 3-OM line, to a level comparable to that in young cells (Fig. 2C). Furthermore, growth curve analysis indicated that all cell lines (both young and old) proliferated better in a cell medium supplemented with MB (dotted lines, Fig. 2D). Together, these results indicated that MB treatment is capable of reducing and/or reversing aging phenotypes in old skin fibroblasts.

### MB upregulates the expression of Nrf2 and its downstream antioxidant genes

The nuclear factor erythroid 2-related factor 2 (Nrf2) is known as an essential regulator of antioxidant defense system by inducing the expression of an array of antioxidant response element (ARE)-containing genes, thereby decreasing overall cellular ROS^22^. A recent study has implicated the Nrf2 antioxidant pathway as a driver mechanism in HGPS^23^. Importantly, MB has been shown to upregulate Nrf2 in neurons^24^.

**Figure 3:**
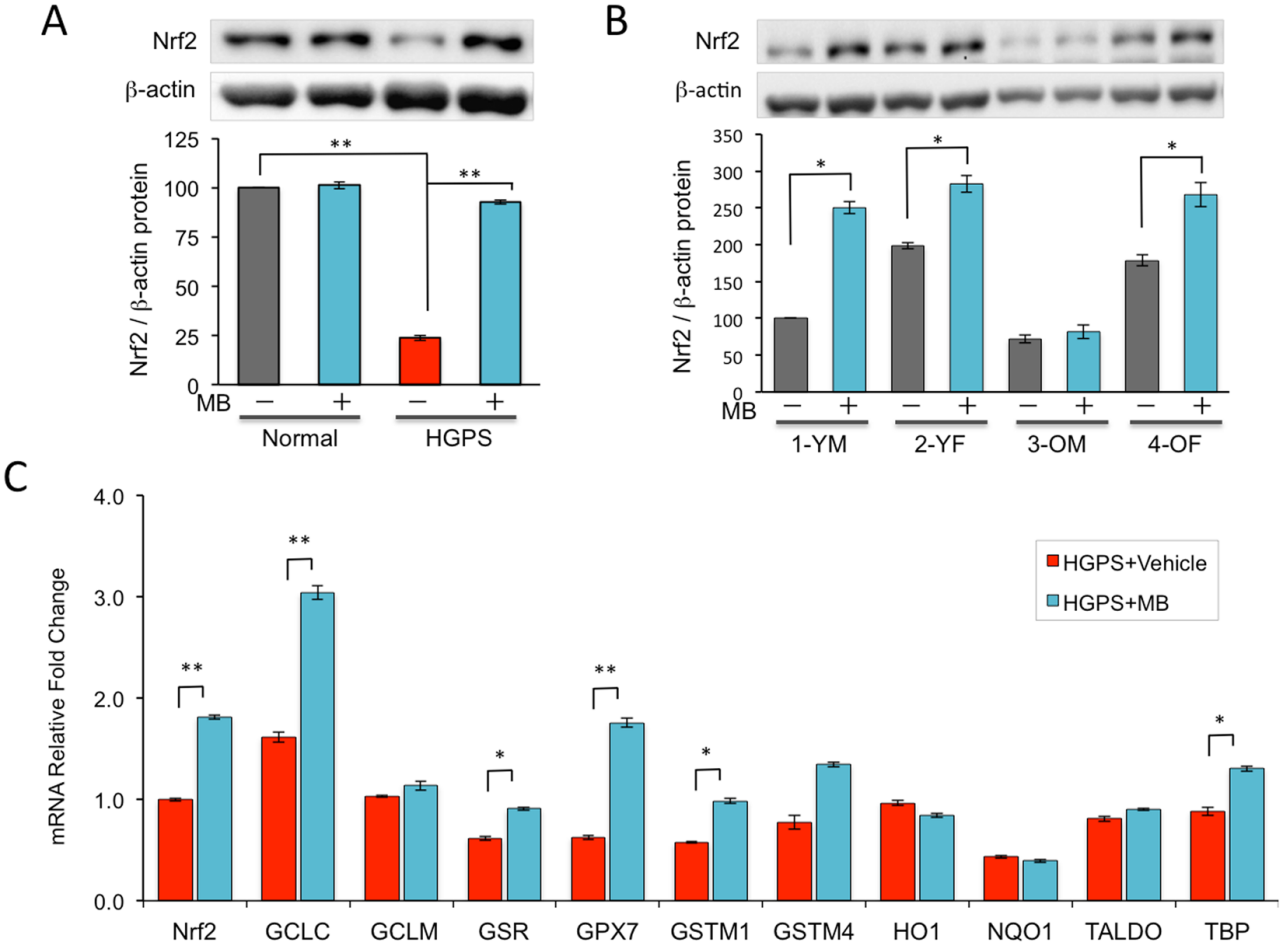
MB upregulates the expression of Nrf2 and its downstream ARE-response genes in human fibroblasts. (**A**) Western blotting analysis showing the changes of Nrf2 protein amounts in normal and HGPS fibroblasts upon four weeks of MB treatment at 100 nM. (**p < 0.01) (**B**) Western blotting analysis showing the changes of Nrf2 protein amounts in the two young and two old fibroblasts after four weeks of treatment upon four weeks of MB treatment at 100 nM. (*p < 0.05) (**C**) Quantitative RT-PCR analysis showing mRNA levels of Nrf2 and its targeting ARE genes in HGPS fibroblasts after four weeks of MB treatment at 100 nM. (*p < 0.05; **p < 0.01).

We speculate that MB may activate the Nrf2-mediated antioxidative response, thereby simulating ROS quenching in the skin fibroblasts. To test this idea, we first examined Nrf2 expression in all six lines of human dermal fibroblasts used in the studies of Figs 1 and 2. Western blotting analysis confirmed the increased Nrf2 protein amounts in most cell lines treated with MB compared to vehicle control (Fig. 3A and B). The old cell 3-OM did not show an obvious increase of Nrf2 protein upon MB treatment probably due to its extreme senescent cell stage thus limited cells for the analysis. Next, we investigated the mRNA levels of Nrf2 downstream ARE-containing genes in HGPS fibroblast line, where the most significant increase of Nrf2 protein upon MB treatment was observed. Quantitative RT-PCR analysis revealed a significant increase in the mRNA expression of a subset of ARE-containing genes, including GCLC, GSR, GPX7, GSTM1, and TBP (Fig. 3C). Together, these analyses support the idea that MB regulates cellular ROS levels at least partially through activating the Nrf2-mediated antioxidative response.

### MB increases tissue viability and shows no signs of irritation on *in vitro* reconstructed 3D human skin

**Figure 4:**
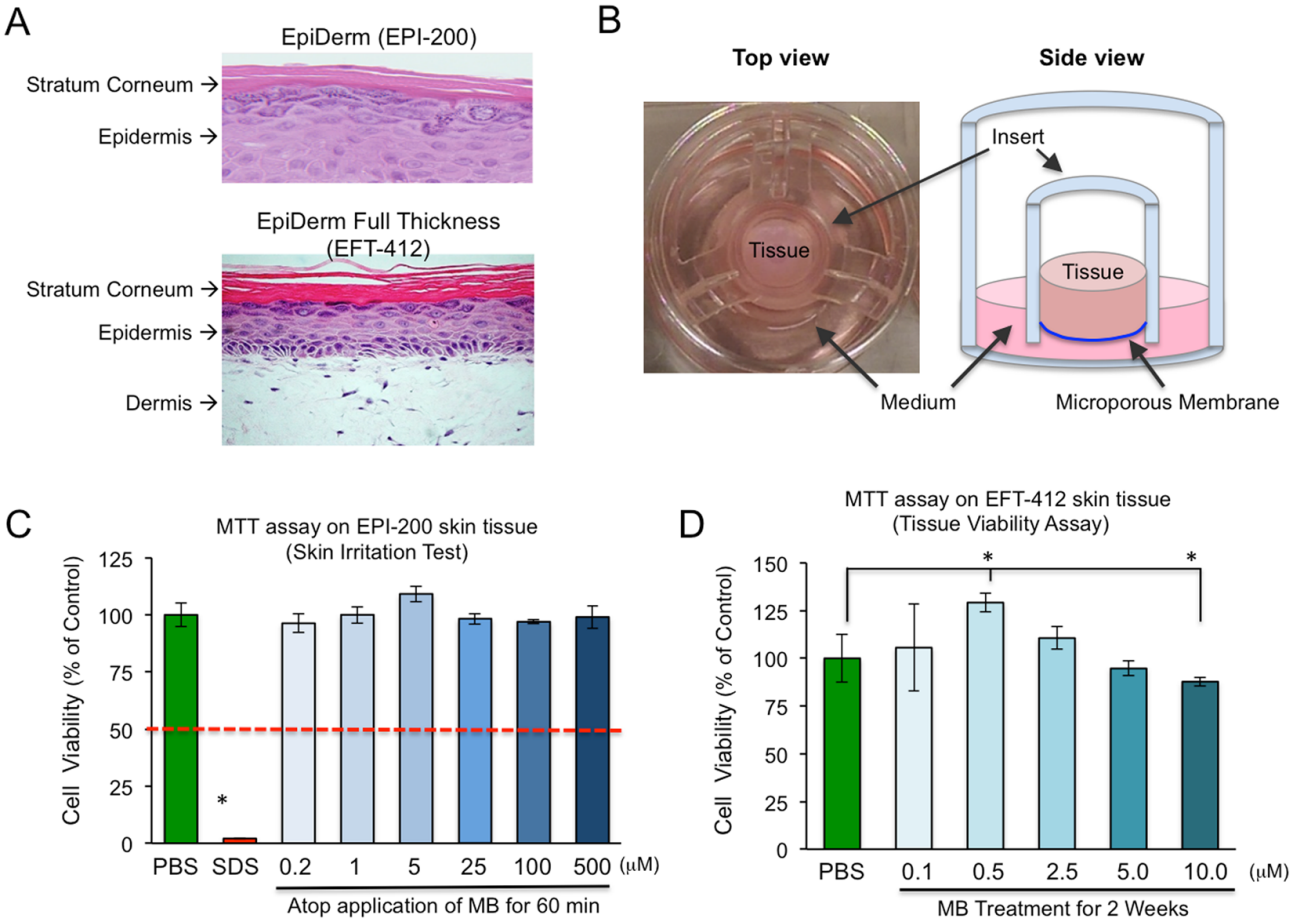
MB increases tissue viability and shows no signs of irritation on the *in vitro* reconstructed 3D human skin. (**A**) H&E staining images showing two kinds of engineered human skin tissues (obtained from MatTek, Ashland, USA). Upper Panel: EpiDerm EPI-200 consists of normal human-derived epidermal keratinocytes (NHEK) and the outer-most stratum corneum layer. This model was only used for the *in vitro* skin irritation test in 3 C. Lower Panel: EpiDerm Full Thickness EFT-412 consists of normal human dermal fibroblasts (NHFB), NHEK and stratum corneum, which was used for most of the 3D skin tissue experiment described in this study. (**B**) Schematic illustrations of top (Left Panel) and side (Right Panel) views of the engineered 3D skin tissue cultured on a microporous membrane insert. (**C**) The short term skin irritation test. MTT assay was conducted on EPI-200 tissues after topical applications of MB in serial doses for 60 minutes. 5% SDS was used as a positive control (strong irritation) and PBS was used as a negative control (no irritation) in this experiment. (**D**) Long term cell viability test. MTT assay was conducted on EpiDerm Full-Thickness (EFT-412) skin tissues that had been treated with MB for two weeks at the indicated concentrations (*p < 0.05).

Based on MB’s potential as a powerful antioxidant in 2D fibroblast lines, we then explored the effects of MB on 3D reconstructed human skin epidermis. We used two available skin models: the EpiDerm skin model EPI-200 (Fig. 4A, upper panel) and the EpiDerm Full Thickness skin model EFT-412 (Fig. 4A, lower panel). These *in vitro* skin models consist of normal human-derived epidermal keratinocytes and fibroblasts cultured at the air-liquid interface on a semi-permeable tissue culture insert (Fig. 4B, details described in Material and Methods), which mimic human normal skin epidermis and are used as approved replacements of Draize rabbits for the *in vitro* skin irritation test (SIT)^18^.

First, we evaluated MB’s safety by conducting the SIT on the model EPI-200. Skin irritation is characterized by a reversible local inflammatory reaction, and MTT cell viability assay is used to estimate the damage caused by the testing irritant. A reduction of MTT over 50% was indicative of skin irritation, as shown by the positive control (5% SDS, Fig. 4C). MB was tested over a wide range of concentrations from 0.2 μM to 500 μM. None of these dosages significantly affected cell viabilities upon a 60-minute topical exposure (Fig. 4C).

To further test the potential irritations of MB after long-term application on the skin, we supplemented MB at different concentrations in culture medium and incubated the full thickness skin model EFT-412 in these media for a total of two weeks. On day 14, the MTT assay was performed. During this two-week incubation period, we noticed the skin tissues treated with high concentrations of MB (5.0 μM and above) started to appear blue after 3 days, suggesting that MB dosage needs to be limited to avoid its colorant side effect on skin appearance. The tissues treated with lower concentrations of MB (from 0.1 μM to 2.5 μM) did not show any tissue coloring. Consistent with the ability of MB to stimulate cell proliferation, we noticed that, at the dosage of 0.5 μM, MB significantly increased cell viability in comparison to the PBS control. In addition, the tissues treated with higher concentrations of MB (5.0 μM and 10.0 μM) showed a reduction in cell viability (Fig. 4D). Based on these results, it can be concluded that low concentrations of MB (less than 2.5 μM) neither irritate nor color skin and are therefore safe for long-term use. As a result, we performed the follow-up studies with MB at concentrations below 2.5 μM.

### MB increases skin thickness and hydration

**Figure 5:**
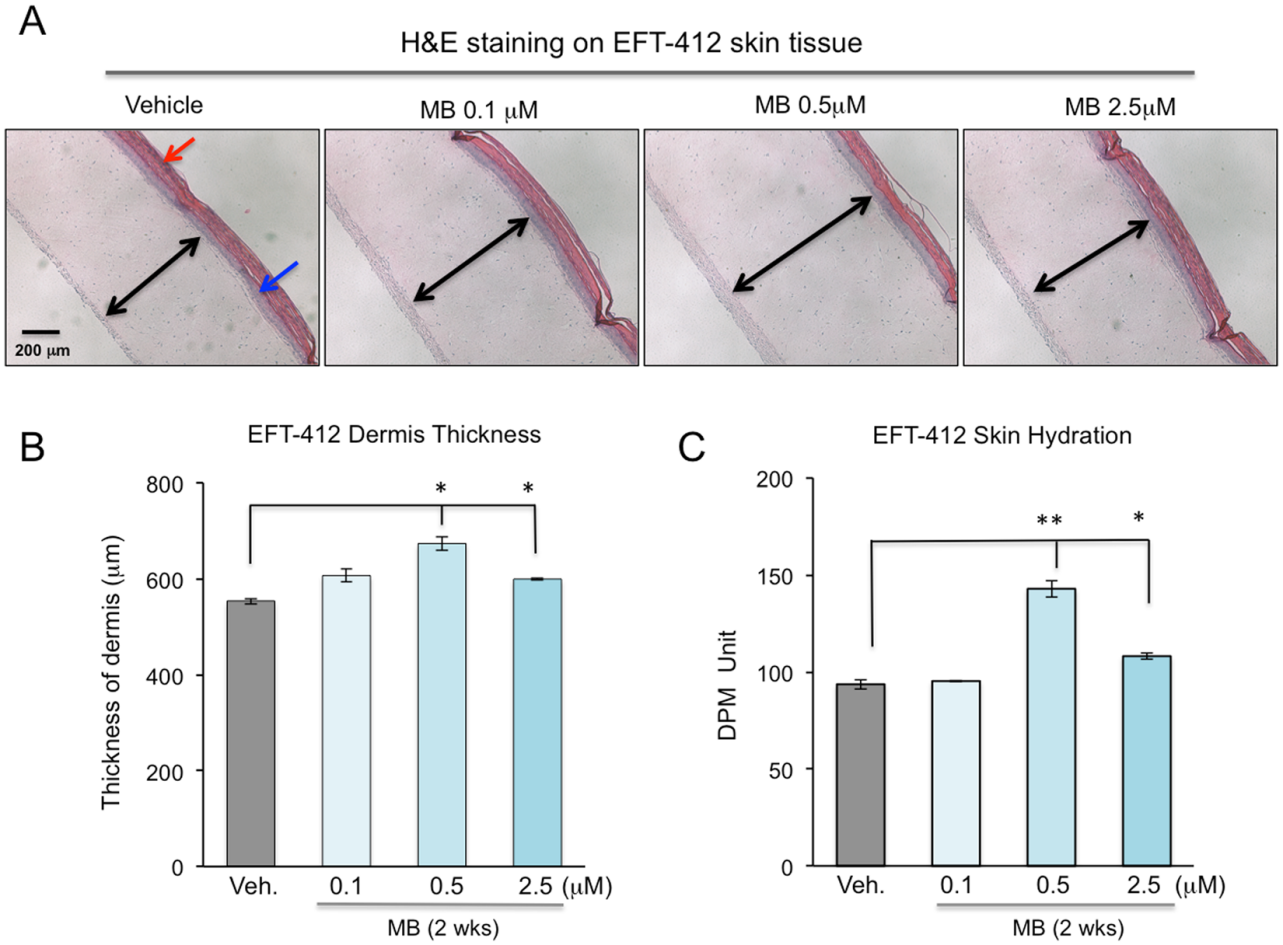
MB increases skin thickness and hydration. (**A**) Representative cross-section images from H&E staining of Epiderm EFT-412 tissues after MB treatment at various concentrations for two weeks. The three skin layers are pointed by colored arrows. Red: the stratum corneum layer, Blue: the keratinocyte layer and black: the dermis fibroblast layer. Scale bar = 200 μm. (**B**) Histogram plot comparing the thickness of fibroblast layer (black arrows in **A**) under different concentrations of MB treatment. The thickness of fibroblast layer was quantified using ImageJ on six consecutive slides for each skin tissue and the average thickness was shown. (**C**) Skin hydration assessed by DPM9003 device showing significantly escalated levels of hydration on the Epiderm EFT-412 skin tissues after two weeks of MB treatment at 0.5 and 2.5 μM. (*p < 0.05, **p < 0.01).

Human skin thickness decreases at an averaged rate of 6% per decade^25^. The gradually thinning skin with age mainly includes a thinner epidermis and dermis, which results in a lowered resistance to shearing forces and higher susceptibility to wounds after trauma^25, 26^. To study the effect of MB treatment on skin thickness, we conducted H&E staining on the EFT-412 skin tissues that had been incubated in culture medium containing 0.1 μM, 0.5 μM or 2.5 μM MB for two weeks, with a fresh replacement of each medium daily. Cross sections of the dermis were then measured after H&E staining (Fig. 5A). We noticed that MB treated skin tissues showed thicker dermis layers than the control PBS-treated dermis (Fig. 5A and B). Quantitative analysis further revealed that the greatest increase in dermis thickness occurred at 0.5 μM MB (Fig. 5B). We also attempted to analyze the thickness of stratum corneum and epidermis layers in those H&E stained skin tissues but found that the thicknesses of these layers were intrinsically highly variable, likely due to tissue preparation and experimental handling.

Human skin retains water mostly through the outermost stratum corneum layer. Loss of hydration in aged skin, due to a decline in function of the stratum corneum, results in a sagging and wrinkling appearance^27, 28^. To study the effect of MB on the stratum corneum layer, we evaluated the water content of the EFT-412 skin tissues that had been incubated in culture medium supplemented with MB for two weeks. In this experiment, the electrical impedances of skin tissues were determined and used as indicators of water content. Consistent with the dermis thickness measurement, we found that skin hydration levels were significantly higher in EFT-412 tissues treated with MB at 0.5 μM and 2.5 μM compared to the PBS control (Fig. 5C). Together, these experiments revealed that MB treatment increases skin dermis thickness and improves skin hydration.

### MB treatment alters the expression of a subset of ECM proteins, including upregulation of elastin and collagen 2A1

**Figure 6:**
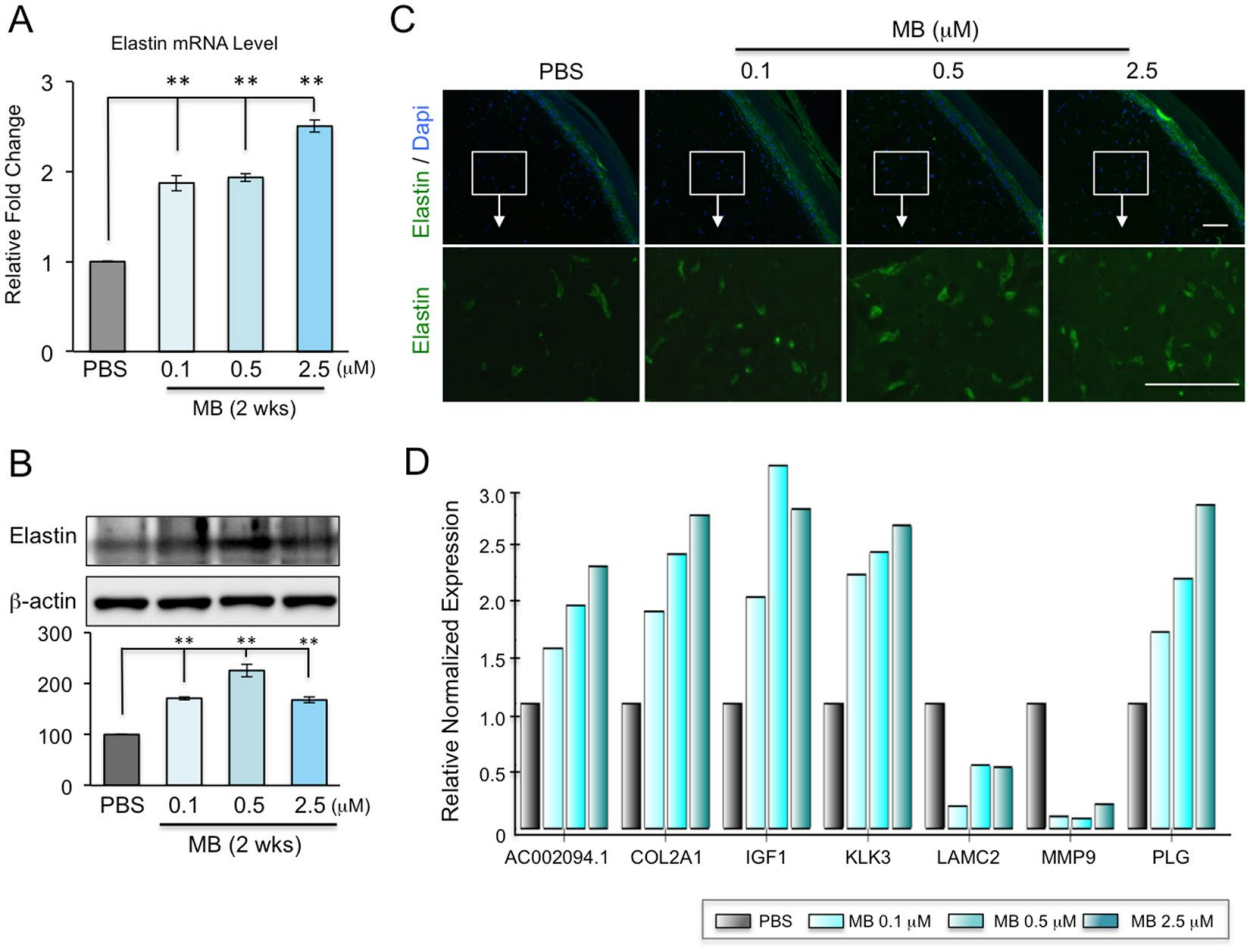
MB upregulates elastin expression and alters other ECM genes expression on 3D skin tissue. (**A**) Quantitative PCR analysis showing significantly upregulated mRNA levels of elastin in EFT-412 skin tissue after two weeks of MB treatment at 0.1, 0.5, or 2.5 μM. (**p < 0.01) (**B**) Western blotting analysis showing increased elastin protein amounts in EFT-412 skin tissues upon two-week treatment of MB at 0.1, 0.5, or 2.5 μM. (**p < 0.01) (**C**) Representative IHC images showing the signals from an anti-elastin antibody (green) on the paraffin slides of 3D skin tissues. Two-week treatment with MB raised elastin signals compared to PBS treatment. Scale bar = 100 μm. (**D**) qPCR analysis showing the significantly up- or down-regulated ECM genes in EFT-412 skin tissues in response to MB treatment compared to PBS treatment.

Elastin, one of the most abundant ECM components in skin dermis, plays an important role in maintaining skin elasticity and resilience. It is synthesized and secreted by dermal fibroblasts and organizes with other ECM proteins into high-order structures^2, 3^. During physiological aging, the elastin production remains relatively stable up to 30~40 years of age then drastically declines afterward^3, 29^. Our previous study indicated that the elastin mRNA level is upregulated by at least two folds in MB-treated normal fibroblasts compared to mock-treated control cells^13^. To test whether this result is transferrable from 2D fibroblast culture to 3D human skin models, we extracted RNA from EFT-412 skin tissues following two-week MB treatment at 0.1, 0.5 and 2.5 μM concentrations. Quantitative RT-PCR analysis revealed that the elastin mRNA levels were significantly increased in the skin tissues treated with MB at all three dosages (Fig. 6A). Western blotting analysis further confirmed the increased elastin protein in MB treated-skin tissues compared to vehicle control (Fig. 6B). Immunohistochemistry with an anti-elastin antibody on the EFT-412 tissue cross sections revealed significantly more elastin fibers in the dermis in 0.5 μM or 2.5 μM MB-treated skin, and a moderate increase of elastin fibers in 0.1 μM MB-treated tissue samples (Fig. 6C).

To further explore whether MB regulates additional ECM components besides elastin, we screened ECM genes using the Bio-Rad PCR array, which contains 30 genes known to be involved in human ECM remodeling. Of these 30 genes, five genes, including COL2A1, IGF1, KLK3, AC002094.1, and PLG, were upregulated by MB and two genes, MMP9 and LAMC2, were downregulated by MB in EFT-412 tissues. Notably, most of these genes showed a dose-dependent response to the concentration of MB (Fig. 6D).

### MB promotes wound healing in dermal fibroblasts

Cutaneous wound healing processes include epidermal keratinocyte migration, dermal fibroblast migration, and the interactions of these cells with the ECM^30^. The skin repair capabilities decline with age due to structural and functional changes, such as reduced proliferation and migration of fibroblasts and degraded collagen and elastin in the ECM^31^. Based on the results from Figs 1–6, we speculate that MB treatment will promote the wound healing of the skin.

**Figure 7:**
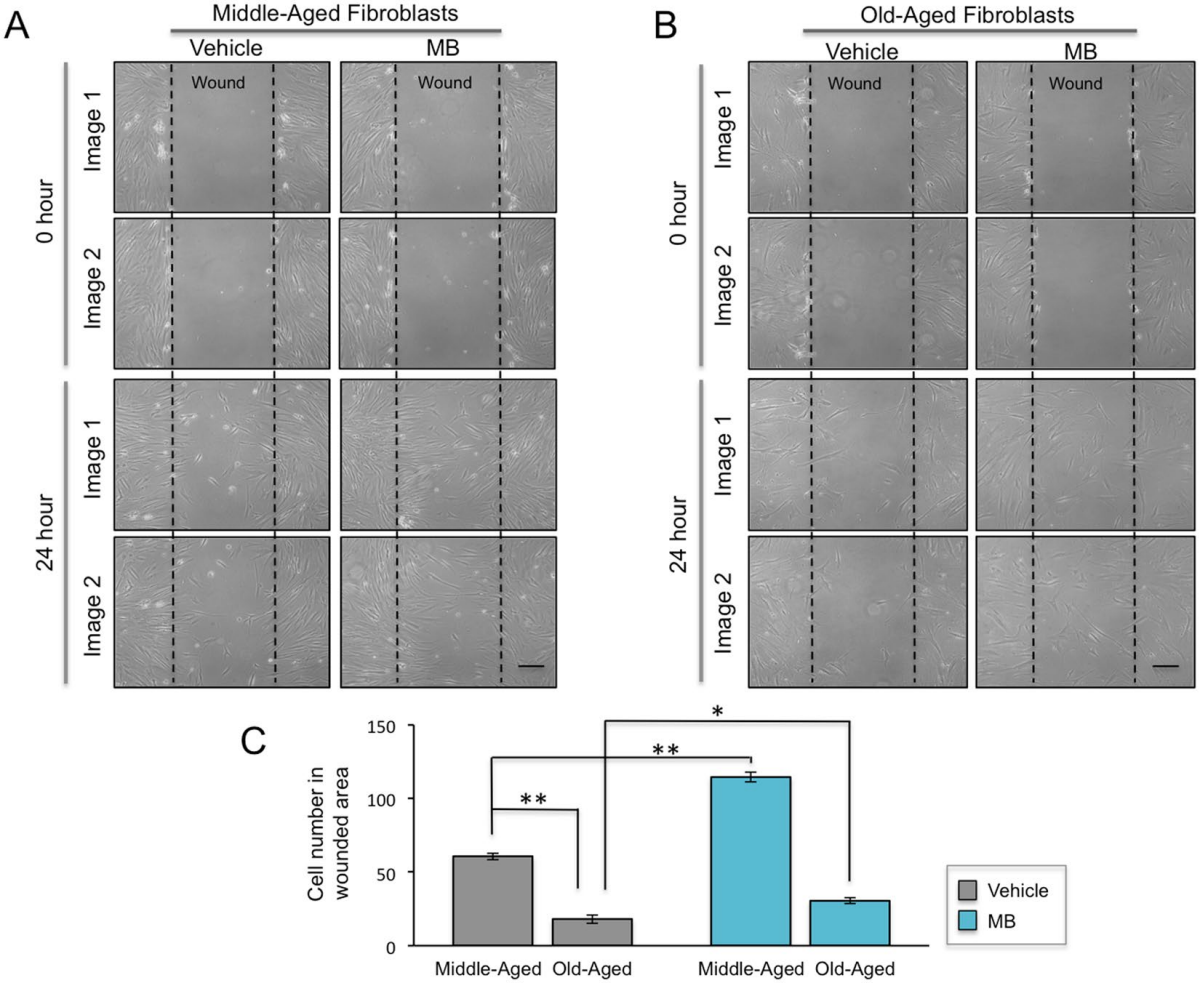
MB promotes wound healing in dermal fibroblasts. (**A**,**B**) Representative images showing skin fibroblasts migration in the scratch-wounded area at 0 and 24 hours post wound. The cells were pre-treated with Vehicle (PBS) or 100 nM MB for one week. The wound region was then manually created by scrapping a straight line across the cultured cells. Fibroblasts from a middle-aged donor (**A**, HGFDFN168, 40 yrs) and an old-aged donor (**B**, AG11725, 84 yrs) were tested. Scale bar = 200 μm. (**C**) Quantification of the cell number in each wounded region at 24 hours post wound. (*p < 0.05, **p < 0.01).

To test this hypothesis, we performed an *in vitro* wound assay, which mimics the cutaneous wound healing process^30, 32^. Fibroblast monolayers were wounded with a scratch and images of cell movement in the scratched area were captured at 0 and 24 hours post wounding. Two normal skin fibroblast lines, one derived from a middle-aged individual and the other from an 84-year old individual, were investigated. As expected, fibroblasts from the middle-aged donor exhibited faster recovery than those from the old donor (Fig. 7A–C). Significantly, the MB-treated fibroblasts in both cell lines repopulated significantly faster than their vehicle-treated counterparts (Fig. 7A–C), suggesting that MB treatment promotes wound healing.

In summary, our analyses using the 2D dermal fibroblasts and 3D reconstructed skin models support the idea that MB is a safe and potent anti-oxidant, and has great potential to be used in skin care.

## Discussion

### MB is a better and safer ROS scavenger than NAC, MitoQ, and mTEM

In our study, we compared MB with three other ROS scavengers, including a widely used general antioxidant NAC, and two mitochondrial-specific antioxidants, MitoQ, and mTEM, and found that MB was the most effective, mTEM was the next best at reducing mitochondrial ROS and promoting cell proliferation (Fig. 1). To our surprise, we observed no beneficial effects of NAC treatment and even adverse effects of MitoQ (Fig. 1). The discrepancy of our results with some previous reports might be due to the much longer-term treatments of NAC or MitoQ in this study than previous experiments^11, 21, 33^. As shown previously^9, 10, 13, 14^ and in Fig. 4, MB has been used in cells and animals for long-term experiments with little toxicity or irritation. Therefore, we suggest that MB is a much safer and more effective antioxidant than NAC, MitoQ, and mTEM for long-term application on skin fibroblasts.

### How does MB reduce ROS and stimulate cell proliferation?

We speculate that MB exerts its potent antioxidant effects through multiple pathways, involving in both blocking oxidant production^10, 34, 35^ and boosting antioxidant defense. MB possesses many unique chemical and physical properties, including a wide solubility in both water and organic solvents (Supplemental Table 2) and a low redox potential^9, 10^. These properties allow MB to penetrate easily through bilayer membranes and reach different cellular compartments like mitochondria and the nucleus, and to exchange swiftly between its oxidized form MB and its reduced form MBH2. When entering mitochondria, MB acts like an alternative electron transporter. It is firstly reduced to MBH2 by the NADH-dehydrogenase of complex I, and then re-oxidized back to MB by cytochrome c. Cycling between those two forms facilitates electron transportation for ATP synthesis, the major function of the mitochondria^9, 10^. More importantly, it prevents electron leakage for oxidants formation, the toxic side products in mitochondria^10^. MB also improves mitochondrial function by inducing PGC1α, a central mediator of mitochondrial biogenesis^13, 35^, and a few key electron transport chain (ETC) components including COX II and COX IV^9, 10, 34^. Overall, these data support that MB promotes mitochondrial function and reduce ROS production.

Interestingly, new emerging data suggest that MB could also influence the central antioxidant defense pathway and other essential functions in the cell, likely through altering gene expression. A previous analysis in neurons^24^ and our study in skin fibroblasts (Fig. 3A and B) demonstrated that MB effectively upregulates Nrf2 expression. Nrf2 controls the basal and induced expression of an array of ARE–dependent genes to sense oxidants and regulates antioxidant defense. Accordingly, we observed a significant increase in the mRNA level of some ARE-dependent genes (Fig. 3C). Previously we conducted an RNA-seq analysis comparing the gene expression profiles in the MB- and mock-treated normal and HGPS fibroblasts^13^. Over one thousand differentially expressed genes were identified in HGPS samples upon the treatment of MB^13^. Among these genes, Gene Ontology (GO) analysis indicates two significantly enriched gene clusters: (1) cell cycle and mitosis and (2) DNA damage response and repair (Supplemental Table 4). In support of the GO analysis results, MB was reported to stimulate robust cell proliferation and delay cellular senescence in many primary cell lines^10, 13, 35^. Moreover, we recently showed that MB treatment promotes DNA damage repair in fibroblasts^36^.

Altogether, these data suggest that MB possesses a wide range of beneficial effects on the fibroblast cells, potentially due to its internal chemical and physical properties and its ability to alter the expressions of key genes involved in antioxidant defense, mitochondrial function, cell cycle and DNA damage repair pathways. Future studies are required to understand how MB alters gene expression in the cells.

### How does MB increase dermis thickness and hydration?

Human skin thickness decreases in an age-dependent manner in both the epidermis and dermis^25^. This time-dependent alteration is caused by the gradual accumulation of cellular ROS, a reduction in the number of skin cells, deficient collagen and elastin, and their disorganization. As a result, skin strength and resiliency are reduced, accompanied with weakened skin barrier function and delayed wound healing^2^. Based on the expression analysis of the ECM components (Fig. 6), we confirm that the increased thickness of the dermis layer produced upon MB treatment (Fig. 5) is at least partially resulting from the increased expression of ECM components, e.g. Col2A1 and elastin, two major structural and functional proteins in the dermis. In addition, the mitochondrial-protective effects by MB may also contribute to energy production required during cell proliferation and maintenance of the dermis^27^.

One of the most important functions of the skin is to provide a barrier to protect the body against environmental insults and to prevent excess water evaporation. This skin barrier function is mainly due to the stratum corneum, which consists of multiple layers of dead corneocytes, and is the final stage of epidermal differentiation^27^. The thickness of the stratum corneum and its intercellular lipid content contribute to the quality of the barrier function. As we age, the skin barrier function declines mainly due to decreased lipid content^37^. It has been reported that MB activates PGC1α which is the coactivator for PPARγ, one of the key regulators in adipogenesis^13, 38^. Thus, we postulate that MB treatment may increase the lipid content of the stratum corneum, leading to increased skin hydration. Additionally, MB treatment was shown to promote K14 expression^13^, which may contribute to a dense network of keratin proteins in stratum granulosum, thereby preventing water evaporation.

### Can MB treatment reduce skin wrinkling?

Skin wrinkling is one of the most visible clinical features in aged skin, which can be exacerbated by exposure to the sun, smoke, or dehydration. The molecular mechanisms underlying skin wrinkling result from a substantial deterioration of the skin matrix molecules, i.e. diminished and disorganized collagen and reduced and distorted elastic fibers^1–3^.

Collagen is the most abundant ECM protein generated by dermal fibroblasts. Type I collagen accounts for about 80% of the dry weight of the dermis and other types (II, III, IV) are also found in skin tissues. Our results showed a dose-dependent increase in COL2A1 at the transcriptional level upon MB treatment (Fig. 6D). We did not observe changes in type I collagen, probably due to its high enrichment in EFT-412 skin tissue that is reconstructed from adult cells. MMPs, the collagen-degrading enzymes secreted by keratinocytes and fibroblasts, promote collagen breakdown and decrease collagen synthesis^2^. In the present study, we found a significant inhibition of MMP9 expression upon MB treatment (Fig. 6D), suggesting an attenuation of collagen degradation in the ECM. Insulin-like growth factor 1 (IGF-1), a hormone secreted by dermal fibroblasts and keratinocytes, upregulates the expression of collagen and inhibits MMP1^39^. The expression of IGF-1 decreases with increasing age^40^. In this 3D skin model, we have shown an upregulation of IGF-1 transcription upon MB treatment (Fig. 6D), which further supports the idea that MB treatment increases collagen in the dermis. Elastin, the second most abundant ECM component, is a fibrous protein that contributes to 2~4% of the dry weight of the dermis. It provides natural elasticity and strength to human skin and also plays a role in tissue repair^3, 41^. We found a robust increase in elastin expression by MB, suggesting that MB treatment enhances skin elasticity and improves skin wound healing (Fig. 6A–C). Based on these results, we speculate that by regulating and orchestrating the expression of these ECM genes, MB may reduce the formation of skin wrinkles. In addition, the enhanced skin hydration from MB application will delay the development of wrinkles.

In summary, MB at nanomolar concentration is potent to scavenge free radicals and stimulate cell proliferation in both young and old dermal fibroblasts. MB treatment on 3D reconstructed skin models provides strong evidence of its potential for improving skin viability, increasing skin hydration and thickness, promoting skin elastin and collagen synthesis, and protecting the skin matrix through the inhibition of enzymatic degradation by MMP. Altogether, our study suggests that MB can be a promising agent for use in anti-aging cosmetics.

## Materials and Methods

### Cell culture and drug treatment

The HGPS and normal human skin fibroblast lines were obtained from the Progeria Research Foundation (PRF) and Coriell Institute (detailed information described in Table S1). The progeria cell line carries the classic C1824T mutation. All fibroblast cell lines were cultured in MEM (Life Sciences) supplemented with 15% FBS (Gemini Bio-Products) and 2 mM L-glutamine (Life Sciences) at 37 °C with 5% CO_2_. Methylene blue (MB, Acros Organics), N-Acetyl-L-cysteine (NAC, Acros Organics), and MitoTEMPO (mTEM, Sigma) were dissolved in PBS and added to the growth medium at a final concentration of 100 nM, 100 μM, or 100 nM respectively. MitoQ (Kindly provided by Dr. Michael P. Murphy) was dissolved in DMSO and added to the growth medium at a final concentration of 100 nM (Table S2). Fresh medium was replaced two or three times a week, and the cultures were passaged 1:3 at 95% confluence.

### Epidermis model and skin irritation test

The reconstructed human epidermis tissues (EpiDerm, EPI-200, Fig. 3A upper panel) were purchased from MatTek Corp (Ashland, USA). According to the MatTek protocol, on the day of receipt, the EPI-200 skins were conditioned by overnight incubation to release transport-stress related compounds.

After pre-incubation, they were transferred to a 24-well plate supplemented with a fresh medium and topically exposed to 30 μl of negative control (PBS), positive control (5% SDS causes keratinocyte death), and MB from 0.2 μM to 500 μM for 60 min at room temperature. The tissues were then thoroughly rinsed off with PBS and transferred to fresh medium. After 24-hour culture, the cell viability assay was conducted by transferring the tissues to 24-well plate containing MTT medium (1 mg/ml) for another 3-hour incubation. The blue formazan salt formed by cellular mitochondria was extracted with isopropanol, and the optical density of the extracted formazan was determined using a spectrophotometer (SpectraMax M5, USA) at 570 nm. Relative cell viability is calculated for each tissue as % of the mean of the negative control tissue. Skin irritation potential of the tested material is predicted if the remaining relative cell viability is below 50%.

### EpiDerm Full Thickness Skin model and related experiments

The EpiDerm Full Thickness Skin tissue EFT-412 (1.0 cm^2^ surface area) consists of normal, human epidermal keratinocytes and dermal fibroblasts (Fig. 3A, Lower Panel). Both types of cells are cultured to form a multilayered model of the human dermis and epidermis that are mitotically and metabolically active. EFT-412 skin tissue is an ideal *in vitro* system to study the anti-aging efficacy of cosmetic ingredients and formulations, and is applied in the following experiments.

1. Tissue viability assay: After overnight pre-incubation, the tissues were transferred to 6-well plates containing 2.5 ml/well fresh medium supplemented with vehicle (PBS) or MB at various concentrations from 0.1 μM to 10 μM. The culture medium was changed daily. After two weeks incubation, tissue viability by MTT assay was performed as described above.
2. Tissue histological analysis: After two weeks incubation with vehicle or MB, the EFT-412 issues were fixed in 10% formalin solution overnight and transferred in PBS solution on next day. The fixed tissues were sent back to MatTek for histological processing and H&E staining. The IHC staining with the anti-elastin antibody was performed on the cross sections following Abcam IHC-paraffin protocol.
3. Hydration test: DPM 9003 device (NOVA, USA) attached to a 4-mm sensor probe was used to measure the impedance of EFT-412 skin tissues. Briefly, after two weeks’ incubation in vehicle- or MB-containing medium, the EFT-412 tissue insert was removed from the 6-well plate and placed on a sterile pad in the culture hood. The tissue surface was pat dried with sterile cotton-tipped swabs and then air-dried for additional two minutes. The impedance of four-quarter circles was measured clockwise with the average value representing the hydration of each tissue.

### Wound healing assay in fibroblasts

An equal number of human dermal fibroblasts from middle-aged and old-aged individuals were seeded in a 100-mm dish and cultured in MEM containing PBS (vehicle) or 100 nM MB. After one or two weeks’ pretreatment, a wounded area in the culture dish was made by scratching a straight line in the monolayer of fibroblasts using a sterile 1000-μl pipette tip. Cells were further cultured for another 24 hours, and images of the wounded area were taken at 0, 24 hours to evaluate fibroblast migration.

### Flow cytometry analysis for mitochondrial ROS

To measure mitochondrial superoxide, cells cultured on 60-mm dishes were incubated with fresh complete medium containing 5 μM MitoSOX Red (Life Technologies, M36008) at 37 °C. After 30 minutes, stained cells were harvested by trypsin digestion and rinsed twice with PBS. Single cell suspensions in 400 μl PBS were prepared for FACS analysis (FACS Canto II; BD). MitoSOX Red was excited by a laser at 488 nm, and the data was collected at 582 ± 21 nm.

### RNA extraction, cDNA synthesis, quantitative RT–PCR

After tissue homogenizing, the total RNA from EFT-412 tissues was extracted with Trizol (Life Sciences) and purified using the RNeasy Mini Kit (Qiagen) according to the manufacturer’s instructions. The RNA yield was determined using the NanoDrop 2000 spectrophotometer. 1 μg of total RNA was converted to cDNA using iScript Select cDNA Synthesis Kit (Bio-Rad). Quantitative RT-PCR was performed in triplicate using SYBR Green Supermix (Bio-Rad) on CFX96 real-time system (C1000 Thermal Cycler; Bio-Rad). The relative mRNA level of a specific gene including elastin (*ELN*), Nrf2 (*NFE2L2*) and its downstream targeting genes is normalized to β-actin, a housekeeping gene served as an internal control. The sequences of all primers used in this study are listed in Supplemental Table 3.

### Western blotting

Whole cell lysates for immunoblotting were prepared by dissolving cells in Laemmli Sample Buffer containing 5% of 2-mercaptoethanol (Bio-Rad). Antibodies used for immunoblotting include p16 (sc-468, Santa Cruz), Nrf2 (sc-722, Santa Cruz), Elastin (Ab21607, Abcam), β-actin (A3854, Sigma-Aldrich).

### Senescence Associated β-Galactosidase activity assay

The assay of SA–β-gal activity was performed according to the manufacturer’s protocol (#9860; Cell Signaling). Briefly, fibroblast cells grown on a six-well plate were fixed in 1X fixative solution containing 2% formaldehyde and 2% glutaraldehyde for 10 minutes and subsequently stained overnight (15 hours) at 37 °C with the β-galactosidase staining solution at pH 6.0 for 15 hours. Images were acquired by Zeiss AX10 microscope with a SPOT PURSUIT camera.

### Statistical Analysis

Results are presented as the mean ± standard deviation. Data were analyzed using 2-tailed Student’s t test, and a *p* value less than 0.05 was considered significant.

## Electronic supplementary material

Supplemental Tables

## References

[1] Binic, I., Lazarevic, V., Ljubenovic, M., Mojsa, J. & Sokolovic, D. Skin ageing: natural weapons and strategies. *Evid Based Complement Alternat Med*. 827248, doi:10.1155/2013/827248 (2013). 10.1155/2013/827248 PMC3569896 23431351

[2] Jadoon, S. *et al*. Anti-Aging Potential of Phytoextract Loaded-Pharmaceutical Creams for Human Skin Cell Longetivity. *Oxid Med Cell Longev* 709628, doi:10.1155/2015/709628 (2015). 10.1155/2015/709628 PMC4581564 26448818

[3] Uitto J (2008). The role of elastin and collagen in cutaneous aging: intrinsic aging versus photoexposure. J Drugs Dermatol.

[4] Poljsak, B., Milisav, I., Lampe, T. & Ostan, I. Reproductive benefit of oxidative damage: an oxidative stress “malevolence”? *Oxid Med Cell Longev* 760978, doi:10.1155/2011/760978 (2011). 10.1155/2011/760978 PMC3182373 21969876

[5] Rinnerthaler M, Bischof J, Streubel MK, Trost A, Richter K (2015). Oxidative stress in aging human skin. Biomolecules.

[6] Masaki H (2010). Role of antioxidants in the skin: anti-aging effects. J Dermatol Sci..

[7] Schirmer RH, Adler H, Pickhardt M, Mandelkow E (2011). Lest we forget you–methylene blue…. Neurobiol Aging.

[8] Paciullo CA, McMahon Horner D, Hatton KW, Flynn JD (2010). Methylene blue for the treatment of septic shock. Pharmacotherapy.

[9] Atamna H, Kumar R (2010). Protective role of methylene blue in Alzheimer’s disease via mitochondria and cytochrome c oxidase. J Alzheimers Dis..

[10] Atamna H (2008). Methylene blue delays cellular senescence and enhances key mitochondrial biochemical pathways. Faseb j.

[11] Wu JJ (2009). Mitochondrial dysfunction and oxidative stress mediate the physiological impairment induced by the disruption of autophagy. Aging (Albany NY).

[12] Rohs R, Sklenar H (2004). Methylene blue binding to DNA with alternating AT base sequence: minor groove binding is favored over intercalation. J Biomol Struct Dyn..

[13] Xiong ZM (2016). Methylene blue alleviates nuclear and mitochondrial abnormalities in progeria. Aging Cell.

[14] Harrison DE (2014). Acarbose, 17-alpha-estradiol, and nordihydroguaiaretic acid extend mouse lifespan preferentially in males. Aging Cell.

[15] Banaclocha MM (2001). Therapeutic potential of N-acetylcysteine in age-related mitochondrial neurodegenerative diseases. Med Hypotheses.

[16] Smith RA, Murphy MP (2010). Animal and human studies with the mitochondria-targeted antioxidant MitoQ. Ann N Y Acad Sci..

[17] Dikalov S (2011). Cross talk between mitochondria and NADPH oxidases. Free Radic Biol Med..

[18] Casas JW (2013). *In vitro* human skin irritation test for evaluation of medical device extracts. Toxicology in vitro: an international journal published in association with BIBRA.

[19] Burtner CR, Kennedy BK (2010). Progeria syndromes and ageing: what is the connection?. Nat Rev Mol Cell Biol..

[20] Moreira PI (2007). Lipoic acid and N-acetyl cysteine decrease mitochondrial-related oxidative stress in Alzheimer disease patient fibroblasts. J Alzheimers Dis..

[21] Lee S (2011). Mitochondrial H2O2 generated from electron transport chain complex I stimulates muscle differentiation. Cell Res..

[22] Ma Q (2013). Role of nrf2 in oxidative stress and toxicity. Annu Rev Pharmacol Toxicol.

[23] Kubben N (2016). Repression of the Antioxidant NRF2 Pathway in Premature. Aging. Cell.

[24] Stack C (2014). Methylene blue upregulates Nrf2/ARE genes and prevents tau-related neurotoxicity. Hum Mol Genet.

[25] Branchet MC, Boisnic S, Frances C, Robert AM (1990). Skin thickness changes in normal aging skin. Gerontology.

[26] Waller JM, Maibach HI (2005). Age and skin structure and function, a quantitative approach (I): blood flow, pH, thickness, and ultrasound echogenicity. Skin Res Technol.

[27] Menon GK, Cleary GW, Lane ME (2012). The structure and function of the stratum corneum. Int J Pharm.

[28] Tagami H (1980). Evaluation of the skin surface hydration *in vivo* by electrical measurement. J Invest Dermatol.

[29] Sephel GC, Davidson JM (1986). Elastin production in human skin fibroblast cultures and its decline with age. J Invest Dermatol.

[30] Hulkower KI, Herber RL (2011). Cell migration and invasion assays as tools for drug discovery. Pharmaceutics.

[31] Gosain A, DiPietro LA (2004). Aging and wound healing. World J Surg..

[32] Liang CC, Park AY, Guan JL (2007). *In vitro* scratch assay: a convenient and inexpensive method for analysis of cell migration *in vitro*. Nat Protoc.

[33] Kageyama Y (2012). Mitochondrial division ensures the survival of postmitotic neurons by suppressing oxidative damage. J Cell Biol..

[34] Atamna H, Mackey J, Dhahbi JM (2012). Mitochondrial pharmacology: electron transport chain bypass as strategies to treat mitochondrial dysfunction. Biofactors.

[35] Atamna H, Atamna W, Al-Eyd G, Shanower G, Dhahbi JM (2015). Combined activation of the energy and cellular-defense pathways may explain the potent anti-senescence activity of methylene blue. Redox Biol..

[36] Zhang H (2016). Loss of H3K9me3 Correlates with ATM Activation and Histone H2AX Phosphorylation Deficiencies in Hutchinson-Gilford Progeria Syndrome. PLoS One.

[37] Boireau-Adamezyk E, Baillet-Guffroy A, Stamatas GN (2014). Age-dependent changes in stratum corneum barrier function. Skin Res Technol..

[38] Xiong ZM, LaDana C, Wu D, Cao K (2013). An inhibitory role of progerin in the gene induction network of adipocyte differentiation from iPS cells. Aging (Albany NY).

[39] Noordam R (2013). Serum insulin-like growth factor 1 and facial ageing: high levels associate with reduced skin wrinkling in a cross-sectional study. Br J Dermatol.

[40] Iranmanesh A, Lizarralde G, Veldhuis JD (1991). Age and relative adiposity are specific negative determinants of the frequency and amplitude of growth hormone (GH) secretory bursts and the half-life of endogenous GH in healthy men. J Clin Endocrinol Metab.

[41] Almine JF, Wise SG, Weiss AS (2012). Elastin signaling in wound repair. Birth Defects Res C Embryo Today.

