# Supplementary material for: Anti-Aging Potentials of Methylene Blue for Human Skin Longevity
Source: Sci Rep. 2017 May 30;7:2475. doi: 10.1038/s41598-017-02419-3 (PMC5449383; doi:10.1038/s41598-017-02419-3)

Supplemental Tables

ANTI-AGING POTENTIALS OF METHYLENE BLUE FOR HUMAN SKIN LONGEVITY

Zheng-Mei Xiong, Mike O'Donovan, Linlin Sun, Ji Young Choi, Margaret Ren, and Kan Cao*

Department of Cell Biology and Molecular Genetics, University of Maryland, College Park, MD 20742, USA

* Corresponding Author:
Kan Cao, Ph.D.
Department of Cell Biology and Molecular Genetics
2114 Bioscience Research Building
University of Maryland
College Park, MD 20742
Phone: 301-405-3016
Fax: 443-524-8098

Supplemental Table 1. Information for Human Fibroblast Cell Lines

Cell line (Name in paper)	Passage Number (Starting → Ending)	Gender	Donor Age	Race	Resource
HGFDFN168 (Normal)	P12 → P18	Male	40 yrs 5 mos	Caucasian	PRF *
HGADFN167 (HGPS)	P12 → P18	Male	8 yrs 5 mos	Caucasian	PRF
AG09266 (1-YM)	P13 → P19	Male	26 yrs	Caucasian	Coriell**
AG08434 (2-YF)	P13 → P19	Female	29 yrs	Caucasian	Coriell
AG11695 (3-OM)	P13 → P19	Male	82 yrs	Caucasian	Coriell
AG11725 (4-OF)	P13 → P18	Female	84 yrs	Caucasian	Coriell

1-YM: 1-Young Male 2-YF: 2-Young Female
3-OM: 3-Old Male 4-OF: 4-Old Female

* Progeria Research Foundation
**Coriell Institute for Medical Research

Supplemental Table 2. Information for Four Antioxidants

Name	Solubility	Concentration
N-Acetyl-L-cysteine (NAC)	Water	1 mM
MitoQ	Lipid	100 nM
MitoTEMPO (mTEM)	Water	100 nM
Methylene Blue (MB)	Water/Lipid	100 nM

Supplemental Table 3. Primer sequences used for quantitative real-time PCR analysis

Gene	Forward Primer (5'->3')	Reverse Primer (5'->3')
ELN (Elastin)	TCTGAGGTTCCCATAGGTTAGGG	CTAAGCCTGCAGCAGCTCCT
NFE2L2 (Nrf2)	AGTGGATCTGCCAACTACTC	CATCTACAAACGGGAATGTCTG
GCLC	CAAGAGAAGGGGGAAAGGAC	GACCTCGGGCAGTGTGAAC
GCLM	TCAGGGAGTTTCCAGATGTC	CAATAGGAGGTGAAGCAATG
GPX7	ACTTCAAGGCGGTCAACATC	GGCAAAGCTCTCAATCTCC
GSR	CCCAAGCCCACAATAGAGG	ACCTGCACCAACAATGACG
GSTM1	GCATGATCTGCTACAATCC	CTTGGGCTCAAATATACGG
GSTM4	CCTTGCTCCCTGAACACTC	GTCGTCACTTCCAACCAAC
HO1	AAGACTGCGTTCCTGCTCAAC	AAAGCCCTACAGCAACTGTCG
NQO1	GGCATTCTGCATTTCTGTG	GGCGTTTCTTCCATCCTTC
TALDO	GGGCCGAGTATCCACAGAAG	GGCGAAGGAGAAGAGTAACG
TBP	CGCCAGCTTCGGAGAGTTC	ACAACCAAGATTCACTGTGGATACA
β-actin	CTGGAACGGTGAAGGTGACA	AAGGGACTTCCTGTAACAATGCA

Supplemental Table 4. Gene ontology and pathway analysis showing enrichment of differentially expressed gene clusters in HGPS cells upon MB treatment

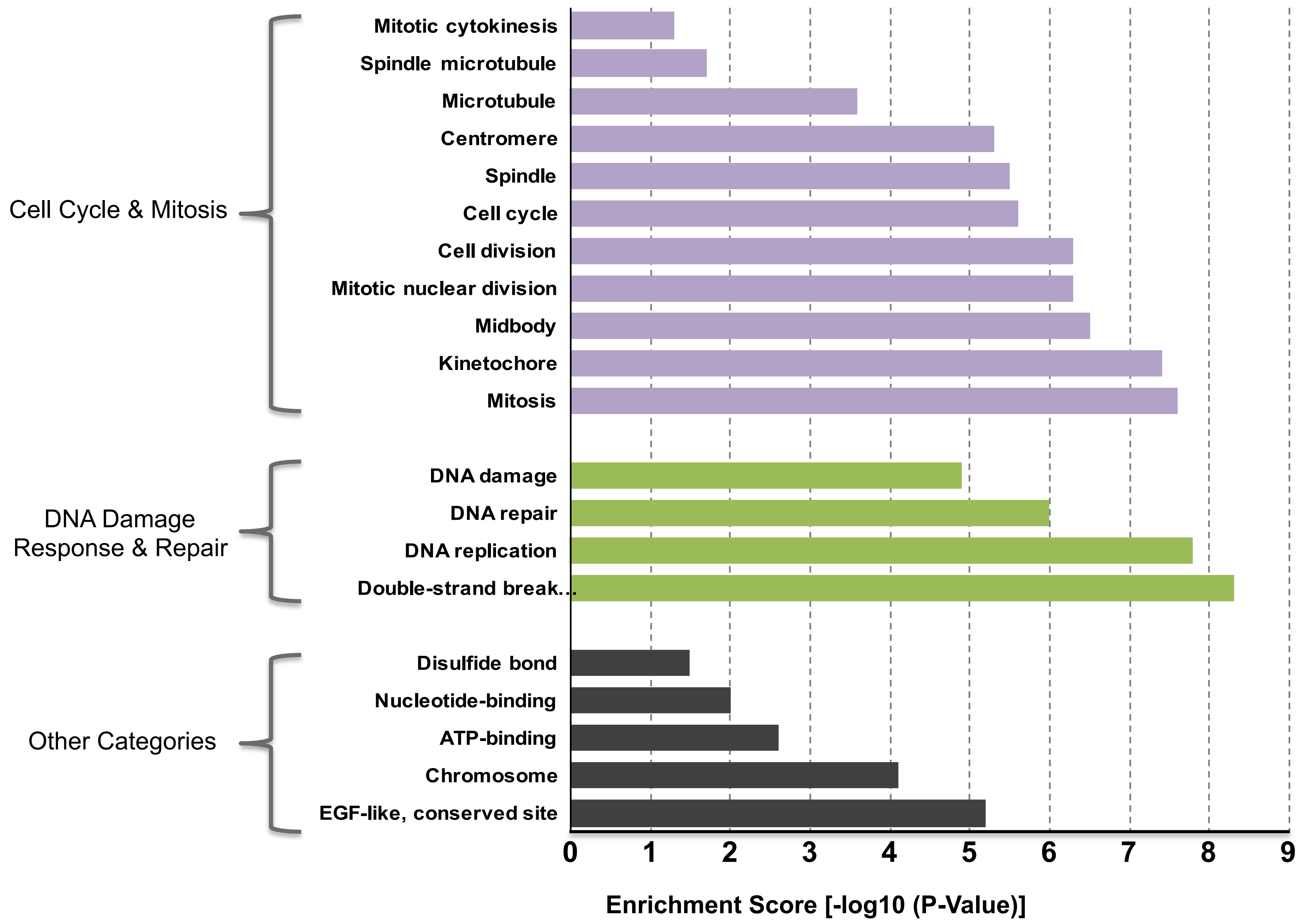

Supplement: Supplementary file 1 — Supplemental Tables [file 41598_2017_2419_MOESM1_ESM.pdf]
